# Transcriptional factor FoxM1-activated microRNA-335-3p maintains the self-renewal of neural stem cells by inhibiting p53 signaling pathway via Fmr1

**DOI:** 10.1186/s13287-021-02191-2

**Published:** 2021-03-10

**Authors:** Jiaoying Jia, Yan Cui, Zhigang Tan, Min Liu, Yugang Jiang

**Affiliations:** Department of Neurosurgery, The Second Xiangya Hospital of Central South University, No. 139, Renmin Middle Road, Furong District, Changsha, 410011 Hunan Province People’s Republic of China

**Keywords:** Neural stem cells, Differentiation, FoxM1, MicroRNA-335-3p, Fmr1

## Abstract

**Background:**

New mechanistic insights into the self-renewal ability and multipotent properties of neural stem cells (NSCs) are currently under active investigation for potential use in the treatment of neurological diseases. In this study, NSCs were isolated from the forebrain of fetal rats and cultured to induce NSC differentiation, which was associated with low expression of the non-coding RNA microRNA-335-3p (miR-335-3p).

**Methods:**

Loss- and gain-of-function experiments were performed in NSCs after induction of differentiation.

**Results:**

Overexpression of miR-335-3p or FoxM1 and inhibition of the Fmr1 or p53 signaling pathways facilitated neurosphere formation, enhanced proliferation and cell cycle entry of NSCs, but restricted NSC differentiation. Mechanistically, FoxM1 positively regulated miR-335-3p by binding to its promoter region, while miR-335-3p targeted and negatively regulated Fmr1. Additionally, the promotive effect of miR-335-3p on NSC self-renewal occurred via p53 signaling pathway inactivation.

**Conclusion:**

Taken together, miR-335-3p activated by FoxM1 could suppress NSC differentiation and promote NSC self-renewal by inactivating the p53 signaling pathway via Fmr1.

## Background

Neural stem cells (NSCs) play a crucial role in the development of the central nervous system, since they can differentiate into astrocytes, neurons, and oligodendrocytes, which are the three major cell types in the central nervous system. NSCs have the ability to continuously self-renew and produce a large number of neuronal and glial lineages [1]. The NSC differentiation capability can potentially restore neurons lost following central nervous system trauma or in neurodegenerative diseases [2]. However, the aberrant differentiation of NSCs may cause central nervous system dysfunction and give rise to certain tumors [3].

Transcriptional factors are highlighted to regulate NSCs biological functions. For instance, PR domain-containing 16 is responsible for NSC maintenance and differentiation [4]. As revealed in previous work, there are as many as 57 transcription-related genes enriched in NSCs, among which forkhead box M1 (FoxM1) is prominent and likely to be essential in the process of NSC differentiation [5]. FoxM1 is a transcriptional factor with a strong involvement in cell proliferation, thus acting as a regulator of differentiation in neuroblastoma cells [6].

MicroRNAs (miRNAs) have been implicated in a gene regulatory network involving neural induction, neuron differentiation, and cell fate specification [7]. Encoded at the genomic region 7q32.2, miR-335 is relevant to the development of various tumors, including human glioma [8], in which miR-335 acts as a pivotal determinant of cell fate [9]. In addition, another study also identified that miR-335 was associated with several malignant tumors of the central nervous system, including glioma and astrocytoma [8, 10]. Although the involvement of a FoxM1-miRNA regulation network in NSCs has been documented [11], it remains unknown how FoxM1 interacts with miR-335-3p to exert effects on NSCs.

Moreover, a prior study elaborated that miR-335 targeted FMRP translational regulator 1 (Fmr1) [12]. Based on previous work, Fmr1 can regulate neuronal migration in developing mouse cortex [13]. Furthermore, there is evidence revealing the diminished p53 expression in Fmr1-knockout adult NSCs [14], whereas activation of the p53 signaling pathway is involved in the self-renewal of mouse NSCs [15]. In our study, we investigated the association between FoxM1-mediated miR-335-3p expression and NSC self-renewal via the p53 signaling pathway under regulation by Fmr1, thus aiming to obtain new insight into the regulatory pathways for differentiation of NSCs.

## Materials and methods

### Ethical statement

The study was conducted with the approval of the Animal Ethics Committee of The Second Xiangya Hospital of Central South University. All the animal experiments were performed in strict accordance with the *Guidelines for the Care and Use of Laboratory Animals* published by the USA National Institutes of Health*.*

### Isolation, characterization, and culture of rat NSCs

The primary NSCs were isolated from the forebrains of fetal rats from pregnant Wistar rats (E14.5; Shanghai Jiesijie Laboratory Animal Co., Ltd., Shanghai, China), following procedures described in a previous study [16]. Under sterile conditions, the cerebral cortex of fetal rats of appropriate gestational age was separated, with careful stripping of the meninges and superficial blood vessels. The cerebral cortex was rinsed 3 times with PBS, and then transferred to 1.5 mL sterile D-Hank solution containing penicillin. Next, the cerebral cortex was cut into blocks (0.5 mm^3^), which were triturated and then allowed to stand for 2 min in a centrifuge tube. The supernatant was gently pipetted into the culture flask, and trypan blue staining was used to count the number of viable cells. NSCs were immersed in NSC basal medium supplemented with NeuroCult proliferation supplement (Stem Cell Technologies, Vancouver, Canada), 1% nitrogen (N2) (Gibco, Rockville, MD, USA), 20 ng/mL basic fibroblast growth factor (BGF; R&D Systems, Minneapolis, MN, USA), 20 ng/mL epidermal growth factor (EGF), and 1% penicillin-streptomycin (Sigma, St. Louis, MO, USA) [17]. The cell density was adjusted to 5 × 10^5^ mL and seeded at 37 °C in an incubator with 5% CO_2_. These cultures were maintained for 3 weeks, and medium of half volume was renewed every week. Spheres that formed during this incubation period were separated by centrifugation at 700 rpm for 30 s. After disaggregation of NSCs by addition of StemPro Accutase cell separation reagent (Gibco), the cells were seeded into uncoated tissue culture plastic dishes at a density of 1 × 10^5^ cells/cm^2^. Neuronal differentiation was induced by culturing the NSCs in NeuroCult™ differentiation medium (Stem Cell Technologies, Vancouver, Canada). The expression of Nestin and SOX2Tuj-1 in cells was assayed by immunofluorescence staining. The proliferation of NSCs was assessed by 5-bromo-2-deoxyuridine (BrdU) assay, and the cellular morphology was observed under an inverted microscope.

### Experimental protocols

NSCs were transfected with different plasmids, including overexpression (oe)-FoxM1, siRNA (si)-FoxM1, miR-335-3p-mimic, miR-335-3p-inhibitor, oe-Fmr1, and si-Fmr1, alone or in combination. Meanwhile, cells were also treated with the inhibitor of p53 signaling pathway (PFT-α; ab120478; Abcam Inc., Cambridge, UK) or dimethyl sulfoxide (DMSO) as control.

The oe-FoxM1 and oe-Fmr1 plasmids were constructed in the pcDNA3.1 vector (Invitrogen, Carlsbad, CA) by Sangon (Shanghai, China). The siRNA expression vector was obtained from the Sigma Mission RNAi shRNA library. In brief, 24 h before the transfection, NSCs were seeded into a 6-well plate (2 × 10^5^ cells/well) and transfected using Lipofectamine 2000 reagent (Invitrogen, Carlsbad, CA) along with 20 nM miRNA, 40 nM siRNA, or 200 ng pcDNA plasmid. At 48 h after this transfection, the cells were collected for subsequent study.

### RNA isolation and quantification

The total RNA was extracted from tissues and cells following the manufacturer’s protocol provided in the TRIzol reagent (15596-018, Beijing Solarbio Science & Technology Co., Ltd., Beijing, China), and its concentration was determined. Then, the total RNA was reversely transcribed into complementary DNA (cDNA; 50 ng/μL) following the instructions of the miRNA reverse transcription kit (D1801; HaiGene, Harbin, China) and the cDNA reverse transcription kit (K1622; Reanta, Beijing, China). The subsequent PCR reaction was performed in a real-time PCR instrument (ViiA 7; Life Technologies, USA). The primers were synthesized by Takara (Liaoning, China) as shown in Table 1. The relative transcription levels were measured using the 2^-△△Ct^ method, with U6 and GAPDH as internal references.

**Table 1:** Primer sequences for RT-qPCR

Genes	Primer sequence (5′-3′)
miR-335-3p	Forward: GTTTTTCATTATTGCTCCTGACCA
	Reverse: CGTGCATCTAGACCGTCATAGA
U6	Forward: ATGACGTCTGCCTTGGAGAAC
	Reverse: TCAGTGTGCTACGGAGTTCAG
FoxM1	Forward: GGCTCCCGCAGCATCAA
	Reverse: TGTTCCGGCGGAGCTCTA
Fmr1	Forward: ATCCCTGCAGAGCACCTCCA
	Reverse: TTCACCTCATGCCCGTGCC
GAPDH	Forward: CTGACATGCCGCCTGGAGA
	Reverse: ATGTAGGCCATGAGGTCCAC

*RT-qPCR*, reverse transcription quantitative polymerase chain reaction; *miR-335-3p*, microRNA-335-3p; *FoxM1*, forkhead box M1; *Fmr1*, FMRP translational regulator 1; *GAPDH*, glyceraldehyde-3-phosphate dehydrogenase

### Western blot analysis

The total protein was extracted from cells following the instructions accompanying the RIPA lysis buffer (R0010; Solarbio, Beijing, China). After extraction of the supernatant, the protein concentration in each sample was detected using a bicinchoninic acid kit (20201ES76; Yeasen, Shanghai, China). Then, the protein was separated by sodium dodecyl sulfate-polyacrylamide gel electrophoresis and transferred onto the polyvinylidene fluoride membrane, which was blocked with 5% bovine serum albumin at room temperature for 1 h. The membrane was then incubated with diluted primary rabbit antibodies (Abcam Inc., Cambridge, UK): FoxM1 (1:500; ab180710), Fmr1-coded FmrR (1 μg/mL; ab17722), Msi1 (1 μg/mL; ab21628), Hes1 (1:500; ab108937), Bmi1 (1 μg/mL; ab38295), Nf-M (1:1000; ab7794), Nestin (1 μg/mL; ab6142), Tuj-1 (1 μg/mL; ab18207), Glial fibrillary acidic protein (GFAP; 1:10000; ab7260), CNPase (5 μg/mL; ab6319), p53 (5 μg/mL; ab26), phosphorylated p53 (1:500; ab1431), p21 (1:1000; ab109199), Fas (ab419, 1:1000), FasL (ab186671, 0.1 μg/mL), and GAPDH (ab9485, 1:500) overnight at 4 °C. Being washed by Tris-Buffered Saline Tween-20 three times (each for 5 min), the membrane was incubated with horseradish peroxidase-conjugated goat anti-rabbit immunoglobulin G antibody (1:20,000; ab205718; Abcam Inc., Cambridge, UK) and developed. The quantitative analysis of protein was conducted using ImageJ 1.48u software (National Institutes of Health, Bethesda, MD, USA) and the relative level was expressed as the ratio of gray value of target band to that of GAPDH band.

### Immunofluorescence staining

The primary NSCs and the differentiated NSCs were fixed by 4% paraformaldehyde for 20 min and penetrated by phosphate buffer saline (PBS)-diluted 0.3% Triton X-100 for 1 h. After being blocked with 10% goat serum, the NSCs were incubated with primary antibodies against Nestin, Tuj-1, and GFAP at 4 °C overnight and then cultured with secondary antibodies for 1 h. The nuclei were counterstained with 1 μg/mL 4′-6-diamidino-2-phenylindole (DAPI). The cells were observed under a Zeiss AX10 microscope (Carl Zeiss, Tornwood, NY, USA) and analyzed using ImageJ (National Institutes of Health, Bethesda, MD, USA).

### BrdU assay

NSC proliferation was determined using the BrdU method following the instructions provided in the commercial kit (Millipore Inc., Bedford, MA, USA). The images were captured using a Zeiss AX10 microscope (Carl Zeiss, Tornwood, NY, USA) and analyzed by ImageJ software.

### Cell Counting Kit-8 (CCK-8)

The sorted cells of each group were seeded into a 96-well culture plate at a density of 1 × 10^3^ cells/well along with 100 μL of a medium containing 10% FBS. The cells were then cultured for 1–5 days and the number of cells was assayed by the CCK-8 kit (Dojindo Laboratories, Kumamoto, Japan) according to the manufacturer’s instructions. In brief, 10 μL CCK-8 solution was added to each well of the plate and incubated for 1 h. The optical density (OD) was measured at 450 nm using a microplate reader.

### Neurosphere formation assay

Neurospheres grown in NSC selective medium were dissociated into single cells by addition of dissociating solution non-enzymatic buffer (C5789, Sigma, St. Louis, MO, USA). The cells were then re-plated at the density of 1–2 cells/mm in a 96-well plate containing stem cell-selective medium. After 6–8 days, the newly formed neurospheres were counted under the microscope, and their size measured (× 200).

### Chromatin immunoprecipitation (ChIP) assay

NSCs were cross-linked with formaldehyde for 10 min and then broken into chromatin fragments (200–1000 bp) using 15 cycles of ultrasonic treatments. Next, the supernatant was incubated with mouse IgG (1:20; 5873S; Cell Signaling Technology, Danvers, MA, USA) as a negative control (NC) and FoxM1 antibody respectively overnight at 4 °C. Subsequently, the protein-DNA complex was precipitated by Pierce protein A/G Magnetic Beads (88803; Thermo Fisher Scientific, Waltham, MA, USA) and de-crosslinked overnight at 65 °C. The DNA was extracted and purified by phenol/chloroform. The primers containing site2 of miR-335-3p promoter which could bind with FoxM1 were designed with the following sequences (5′-3′): Forward: CTCCTGGTCTCTCCCCTCAA and Reverse: GCTGAAACCTACACGACCCA. Meanwhile, the other pair of primers, which could amplify the sequence distal the promoter region of miR-335-3p, was designed to serve as NC, using the following sequences (5′-3′): Forward: CCTGCTCTGCACTCATGGAA and Reverse: TGAAACCTACACGACCCACG. qPCR was then performed with the purified DNA fragments as the amplification template, and with site2 primers and NC primers, respectively, to verify that the site2 of miR-335-3p was indeed the binding site of FoxM1.

### RNA pull-down assay

The binding relationship between miR-335-3p and Fmr1 was examined using a Magnetic RNA-Protein Pull-Down kit (20164; Pierce, Rockford, IL, USA). NSCs were collected and lysed in RIP lysis buffer. Next, the cell lysate was incubated with the biotinylated miR-335-3p, miR-335-3p NC, and streptavidin-conjugated beads at 4 °C overnight. Finally, the RNA was extracted using TRIzol reagent and RT-qPCR was performed to measure the level of Fmr1 mRNA.

### Dual-luciferase reporter assay

The targeting relationship between miR-335-3p and Fmr1 as well as FoxM1 and miR-335-3p was predicted according to biological websites (http://www.targetscan.org; https://cm.jefferson.edu/rna22/Interactive), which was confirmed by dual-luciferase reporter assay. Based on the sequence of miR-335-3p binding to Fmr1, we designed Fmr1 WT and MUT sequences. The 3′UTR gene fragments of Fmr1 WT (vector expressing wild-type Fmr1) and Fmr1 MUT (vector expressing mutant Fmr1) were artificially synthesized and introduced into the PGLO-control vector (Promega, USA) using the XhoI and BamH I endonuclease sites. After restriction endonuclease digestion, we used T4 DNA ligase to insert the target fragments into the PGLO-control vector to construct the desired Fmr1 WT and Fmr1 MUT plasmids. These reporter plasmids were then co-transfected with miR-335-3p mimic and NC mimic plasmids into 293T cells. Twenty-four hours after transfection, cells were lysed and centrifuged at 12,000 rpm for 1 min, followed by the collection of supernatant. Next, the luciferase activity was measured by the Dual-Luciferase® Reporter Assay System (E1910; Promega, Madison, WI, USA). The relative luciferase activity was expressed as the ratio of Firefly luciferase to Renilla luciferase.

Three sites by which FoxM1 was most likely to bind to the miR-335-3p promoter were predicted based on the UCSC (http://genome.ucsc.edu) and JASPAR (http://jaspar.genereg.net) websites: Position 45–51 of FOXM1 three-prime untranslated region (3′UTR): GUCCACCAUCCCGGGCAGGGCAA; Position 56–62 of FOXM1 3′UTR: CUGGCACUUGUGUGGCGUUAGGU; Position 819–825 of FOXM1 3′UTR: UACAAGCUACAGAACAACGGAAC. Next, the recombinant WT and MUT luciferase reporter plasmids were constructed and co-transfected with FoxM1 into 293T cells, to verify the binding relationship between FoxM1 and miR-335-3p promoter. The dual-luciferase reporter assay was carried out following the same procedures as described above.

### Flow cytometry

The cell cycle was analyzed using flow cytometry. NSCs were seeded into 100-mm culture dishes with the density of 1 × 10^6^ cells/mL, and cultured at 37 °C with 5% CO_2_. After 36–48 h, cells were collected and then washed with PBS for 2–3 times and then resuspended in PBS at a density of 1 × 10^6^ cells/mL. Next, cells were fixed by addition of 0.5 mL of pre-cooled 70% ethyl alcohol overnight at − 20 °C and stored on ice for 1 h, followed by 2-min centrifugation at 4000 rpm. Subsequently, cells were precipitated, resuspended in 0.5 mL PBS containing 0.25% Triton X-100, and then incubated on ice for 15 min, and centrifuged at 4000 rpm for 2 min. After discarding the supernatant, cells were resuspended in 0.5 mL PBS containing 10 μg/mL RNase A and 20 μg/mL PI stock solution (P4170; Sigma-Aldrich, St. Louis, MO, USA), and incubated at room temperature in dark for 30 min. Finally, the centrifuged cells were filtered through a 300-μM nylon net filter into Eppendorf tubes containing PBS. The cell cycle of the filtered cells was analyzed using the FACS Aria III system (BD Biosciences, San Jose, CA, USA).

### Statistical analysis

The experimental data in this study were processed using SPSS 21.0 statistical software (IBM Corp., Armonk, NY, USA) and presented as mean ± standard deviation. The number of neurospheres was calculated under light microscopy in 15 randomly-selected, non-overlapping fields. If data were normally distributed with homogeneity of variance, comparison between two groups was analyzed by unpaired *t* test, differences among multiple groups were analyzed by one-way analysis of variance (ANOVA) or repeated measures ANOVA, and the pairwise comparison within group was conducted using post hoc test. Otherwise, the data were analyzed using rank-sum test. When the *p* value was less than 0.05, the difference was considered statistically significant.

## Results

### miR-335-3p was downregulated during NSC differentiation

**Fig. 1:**
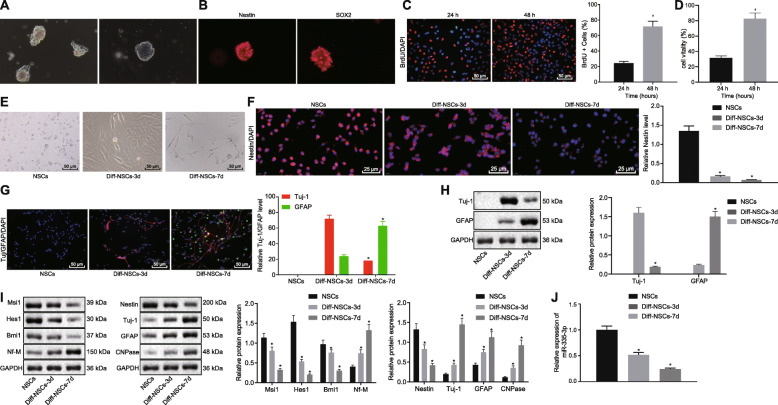
Downregulation of miR-335-3p was found during NSCs differentiation. **a** Microscopic views of neurosphere formed by the in vitro subculture of NSCs. **b** Nestin and SOX2 expression (red) in neurosphere detected by immunofluorescence assay. **c** NSC proliferation activity determined using BrdU assay (× 200). **d** The cell viability of NSCs assessed by CCK-8 assay. **e** NSC morphology after induced-differentiation observed on days 0, 3, and 7 (× 200). **f** Nestin expression (red) in NSCs after induced-differentiation measured using immunofluorescence assay on days 0, 3, and 7 (× 400). **g** Tuj-1 and GFAP expression in NSCs after induced-differentiation examined by immunofluorescence assay on days 0, 3, and 7 (× 200). **h** Western blot analysis for the expression of Tuj-1 and GFAP in NSCs after induced-differentiation on day 0, 3, and 7. **i** Western blot analysis of expression of Msi1, Hesl, Bmi1, Nf-M, Nestin, Tuj-1, GFAP, and CNPase in NSCs. **j** Expression of miR-335-3p assayed by RT-qPCR in NSCs after induced-differentiation on days 0, 3, and 7. The data in **c** and **d** were enumeration data, while the data in **f**–**h** were measurement data described as mean ± standard deviation. Unpaired *t* test was used for the comparison between two groups, while one-way ANOVA was employed for the analysis data among multiple groups, followed by Tukey’s post hoc test. *p < 0.05 versus NSCs without any induced-differentiation treatment or NSCs with induced-differentiation treatment at 24 h. The experiment was repeated in triplicate

To establish the expression rate of miR-335-3p in NSCs, primary NSCs were first isolated from the forebrains of fetal rats delivered from pregnant Wistar rats. After 3 days of subculture in vitro, NSCs stably grew as neurospheres, while NSC medium maintained dry. After 15 days, we found that the neurospheres were enlarged, but without cell adherence and protrusions (Fig. 1a). Meanwhile, immunofluorescence assay revealed that the expression of Nestin and SOX2 was positive in these neurospheres (Fig. 1b). According to BrdU and CCK-8 assays, the separated NSCs possessed strong proliferative ability (Fig. 1c, d). Therefore, NSCs were successfully separated.

To verify the differentiation ability of the isolated NSCs, in vitro differentiation of NSCs was obtained by culturing in NeuroCult differentiation medium. As displayed in Fig. 1e, the NSCs were spherical with a clear boundary and high refractive index, and their surface was smooth, without protuberances. After 3 days of culture in the differentiation medium, the neurospheres started to differentiate, and 7 days later, neurites had appeared and synaptic connections formed between the cells, indicating that the NSCs had differentiated.

Next, immunofluorescence assay results showed that with increasing duration of induced-differentiation, the expression of Nestin decreased gradually, becoming significantly reduced from the 3rd day of differentiation onward (*p* < 0.05) (Fig. 1f). Meanwhile, NSCs were mainly differentiated into neuron-type cells on the 3rd day but astrocytes on the 7th day, as reflected by the elevated expression of Tuj-1 and GFAP (Fig. 1g). In addition, Western blot assay showed that, compared to the expression on day 3, the cellular expression of Tuj-1 was significantly lower and that of GFAP was significantly increased on day 7 (Fig. 1h). Thus, NSCs mainly differentiated into neurons in the early stage and later mainly differentiated into glial cells. As exhibited by the above results, we had successfully isolated NSCs from the rats, which showed characteristics of multilineage differentiation and self-renewal capabilities under appropriate conditions.

We next conducted Western blot analysis to detect the cellular expression of self-renewal-related genes (Msi1, Hes1, Bmi1, Nf-M) [18] and differentiation-related genes (Nestin, Tuj-1, GFAP, CNPase) [19], as these markers are related to neural stem cell proliferation and differentiation. With the increase of differentiation time, the expression of Msi1, Hes1, Bmi1, and Nestin decreased, while the expression of Nf-M and Tuj-1, GFAP, and CNPase increased (Fig. 1i). Additionally, based on previous work showing that miR-335-3p was implicated with NSCs self-renewal [20], we detected the expression of miR-335-3p at different time points during NSC differentiation using RT-qPCR. Results showed that the level of miR-335-3p was reduced during NSC differentiation (Fig. 1j). Taken together, these results indicated that miR-335-3p might function as a modulator of NSC self-renewal.

### Upregulation of miR-335-3p maintained self-renewal of NSCs

**Fig. 2:**
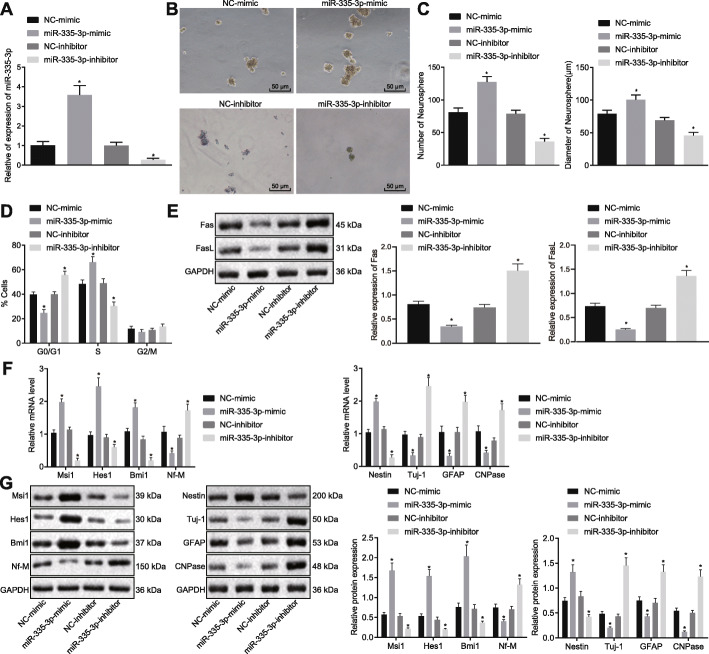
Overexpressing miR-335-3p repressed NSC differentiation but maintained NSC self-renewal. NSCs were treated with NC-mimic, miR-335-3p mimic, NC-inhibitor, or miR-335-3p inhibitor. **a** Expression of miR-335-3p in NSCs detected by RT-qPCR. **b** Microscopic observation of neurosphere formation ability (day 7) (× 200). **c** Quantitative analysis for number and size of neurospheres. **d** Cell cycle distribution of NSCs measured by flow cytometry. **e** Western blot analysis for the expression of Fas and FasL. **f** Expression of self-renewal-related and differentiation-related genes assayed by RT-qPCR. **g** Representative protein bands and corresponding histograms of the expression of self-renewal-related and differentiation-related genes tested by Western blot analysis. The measurement data were described as mean ± standard deviation. Data comparisons among multiple groups were conducted by one-way ANOVA, followed by Tukey’s post hoc test. *p < 0.05 versus NC-mimic or NC-inhibitor. The experiment was conducted three times

Aiming to explore the role of miR-335-3p in the self-renewal and differentiation of NSCs, we introduced miR-335-3p mimic and miR-335-3p inhibitor into NSCs (Fig. 2a). After performing neurosphere formation assay, we found that the number and size of neurosphere formed from NSCs treated with miR-335-3p mimic were significantly increased, whereas miR-335-3p inhibitor had the opposite effect (Fig. 2b, c). Meanwhile, cell cycle distribution of NSCs after different treatments was determined using flow cytometry. As documented in Fig. 2d, miR-335-3p overexpression led to augmented viability of NSCs, characterized by increased proportion of cells arrested in S phase and declined proportion of those in G_0_/G_1_ phase, whereas the opposite results were achieved in response to miR-335-3p inhibition. Fas ligand (FasL) and its receptor Fas are known membrane surface molecules related to cell apoptosis [21]. Herein, we conducted Western blot analysis to detect Fas and FasL expression in an examination of the effect of miR-335-3p on neural stem cell apoptosis. We found that transfection of miR-335-3p mimic resulted in diminished expression of Fas and FasL and reduced apoptosis of NSCs, whereas miR-335-3p inhibitor transfection promoted the expression of Fas and FasL as well as NSC apoptosis (Fig. 2e). These data indicated that miR-335-3p might enhance self-renewal of NSCs and reduce apoptosis. Furthermore, miR-335-3p mimic led to upregulated expression of NSC self-renewal-promoting genes including Msi1, Hes1, and Bmi1, and downregulated expression of Nf-M, which inhibited NSC self-renewal; it also resulted in augmented Nestin expression as well as repressed expression of Tuj-1, GFAP, and CNPase, whereas miR-335-3p interference led to the opposite results (Fig. 2f, g). To sum up, upregulating miR-335-3p was conductive to maintaining the self-renewal of NSCs and to suppressing NSC differentiation.

### Transcriptional factor FoxM1 activated miR-335-3p

**Fig. 3:**
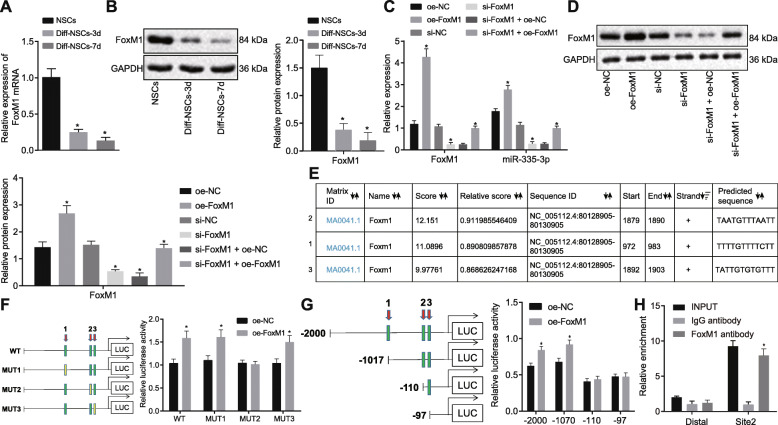
The expression of miR-335-3p was activated by FoxM1. **a** FoxM1 mRNA level in NSCs after induced differentiation detected using RT-qPCR on days 0, 3, and 7. **b** FoxM1 protein level in NSCs after induced differentiation determined by Western blot analysis on days 0, 3, and 7. **c** FoxM1 and miR-335-3p expression in NSCs after alteration of FoxM1 examined using RT-qPCR. **d** FoxM1 protein level in NSCs after alteration of FoxM1 measured by Western blot analysis. **e** 3 sites by which FoxM1 was most likely to bind to miR-335-3p promoter region predicted by online websites. **f**, **g** Dual-luciferase reporter assay for mutant miR-335-3p promoter recombinant luciferase reporter gene vector (**f**) and truncated miR-335-3p promoter recombinant luciferase reporter gene vector (**g**) co-transfected with FoxM1 expression vector into NSCs to determine the targeting relationship of FoxM1 and miR-335-3p. **h** The binding relationship between FoxM1 and miR-335-3p measured using CHIP assay. The measurement data were expressed as mean ± standard deviation. The unpaired *t* test was employed to analyze the differences between two groups, while differences among multiple groups were analyzed using one-way ANOVA, with Tukey’s post hoc test. *p < 0.05 versus NSCs without any induced-differentiation treatment, NSCs treated with oe-NC, si-NC, or si-FoxM1 + oe-NC, or IgG antibody. The experiment was performed at least 3 times

According to prior research, FoxM1 can mediate multiple miRNAs such as miR-130b and miR-301a in NSCs [11]. Moreover, FoxM1 expression was diminished during NSCs differentiation in mouse embryo [22]. Therefore, we first examined the expression of FoxM1 in NSCs. As revealed by RT-qPCR and Western blot analysis, FoxM1 expression was conspicuously decreased in NSCs with the increasing duration of induced-differentiation (Fig. 3a, b), which was consistent with the changes in miR-335-3p expression. Next, we found that overexpressing FoxM1 resulted in elevated miR-335-3p expression, while silencing FoxM1 decreased miR-335-3p expression. When FoxM1 expression was restored after FoxM1 knockdown, miR-335-3p expression was also accordingly increased (Fig. 3c, d).

The above phenomena illustrate that FoxM1 positively regulates miR-335-3p expression. Therefore, we hypothesized that FoxM1 might be able to bind to the miR-335-3p promoter region. To test our prediction, we first predicted from bioinformatics that FoxM1 was likely to bind to 3 sites of the miR-335-3p promoter region, according to the UCSC (http://genome.ucsc.edu) and JASPAR (http://jaspar.genereg.net) websites (Fig. 3e). Next, we performed the dual-luciferase reporter assay (Fig. 3f, g), which showed that site2 was the specific binding site of FoxM1 protein on the miR-335-3p promoter region. Then, we substantiated through ChIP assay that FoxM1 bound to miR-335-3p promoter region at site2 (Fig. 3h). Specifically, the primers covering site2 could amplify more DNA expression when using chromatin fragments precipitated by FoxM1 antibody as the template, relative to control results with IgG antibody (*p* < 0.05). Besides, there was no significant difference in the amounts of amplification products of the two primer pairs in the IgG antibody group (*p* > 0.05). These results concurred in suggesting that the site2 was indeed the binding site of the transcriptional factor FoxM1 in miR-335-3p promoter region.

### FoxM1 inhibited NSC differentiation and induced NSC self-renewal by activating miR-335-3p

**Fig. 4:**
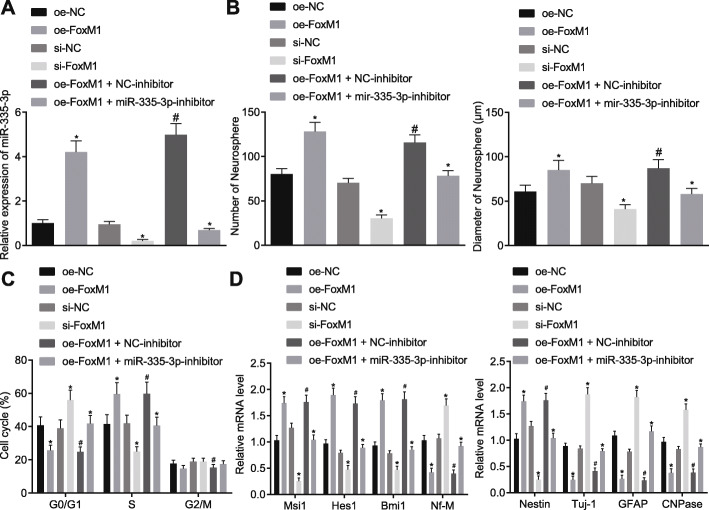
NSC self-renewal was promoted by FoxM1-activated miR-335-3p. NSCs were treated with oe-NC, oe-FoxM1, si-NC, si-FoxM1, oe-FoxM1 + NC-inhibitor, and oe-FoxM1 + miR-335-3p inhibitor. **a** miR-335-3p expression in NSCs detected by RT-qPCR. **b** Statistical analysis of the number and size of neurospheres. **c** NSC cell cycle measured by flow cytometry. **d** Expression of self-renewal-related and differentiation-related genes in NSCs examined by RT-qPCR. The measurement data were expressed as mean ± standard deviation. Data analyses among multiple groups were performed by one-way ANOVA, followed by Tukey’s post hoc test. *p < 0.05 versus oe-NC or si-NC or oe-FoxM1 + NC-inhibitor. ^#^*p* < 0.05 versus oe-FoxM1 + NC-inhibitor and oe-NC. The experiment was performed three times

Since FoxM1 was found to activate the expression of miR-335-3p, in the next step, we attempted to verify whether FoxM1 could modulate NSC self-renewal and differentiation via miR-335-3p. According to RT-qPCR results, overexpressing FoxM1 activated miR-335-3p in NSCs, which was blocked by co-treatment of miR-335-3p inhibitor (Fig. 4a). Then, as shown by the neurosphere formation assay, the number and size of neurosphere in NSCs were increased by overexpressing FoxM1, which was reversed by treatment with miR-335-3p inhibitor. However, the number and size of neurosphere from cells treated with si-FoxM1 were reduced (Fig. 4b). Meanwhile, based on the flow cytometry results, overexpressing FoxM1 increased the proportion of cells at the S phase but decreased cells at G_0_/G_1_ phase, which was abrogated by the simultaneous transfection of both oe-FoxM1 and miR-335-3p inhibitor. Instead, silencing FoxM1 reduced the abundance of cells at the S phase, but elevated cells at the G_0_/G_1_ phase (Fig. 4c).

Furthermore, expression of self-renewal-related and differentiation-related genes was measured by RT-qPCR. An obvious elevation in the expression of Nestin, Msi1, Hes1, and Bmi1 as well as a decrease in that of Nf-M, Tuj-1, GFAP, and CNPase were observed in the presence of FoxM1 overexpression, the effects of which were then abrogated by miR-335-3p inhibition. In contrast, silencing FoxM1 resulted in the opposite tendency of these genes (Fig. 4d). Collectively, the above results suggested that FoxM1 could maintain NSC self-renewal and inhibit NSC differentiation through miR-335-3p.

### miR-335-3p overexpression repressed NSC differentiation and promoted NSC self-renewal by inhibiting p53 signaling pathway via Fmr1

**Fig. 5:**
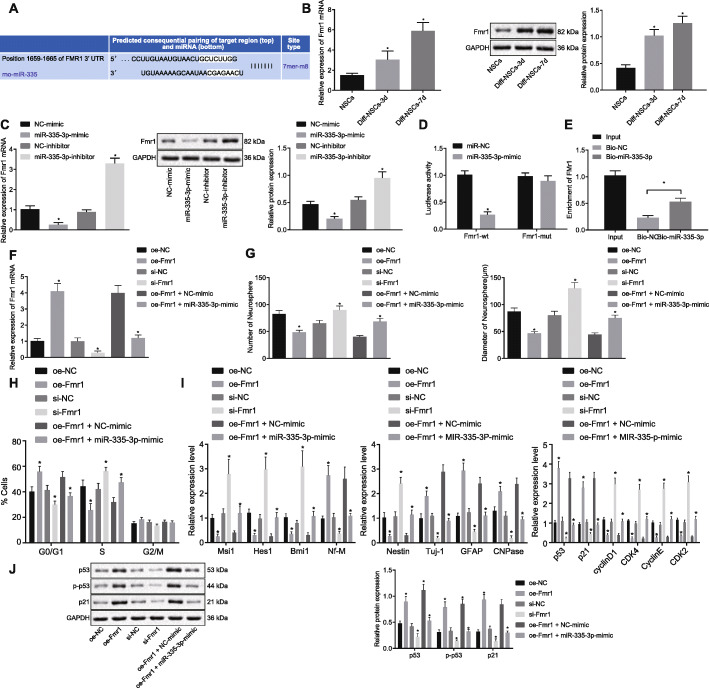
miR-335-3p inactivated p53 signaling pathway, repressed NSC differentiation, and promoted NSC self-renewal by targeting Fmr1. **a** The binding sites between miR-335-3p and Fmr1 3′-UTR predicted by bioinformatics analysis. **b** Fmr1 expression in NSCs after induced differentiation detected on days 0, 3, and 7 by RT-qPCR and Western blot analysis. **c** Fmr1 expression determined after alteration of miR-335-3p in NSCs using RT-qPCR and Western blot analysis. **d** Interaction between miR-335-3p and Fmr1 verified using dual-luciferase reporter assay. **e** The binding relationship between miR-335-3p and Fmr1 attested by RNA pull-down. **f** Fmr1 expression in NSCs after overexpression of miR-335-3p and Fmr1 measured by RT-qPCR and Western blot analysis. NSCs were treated with oe-NC, oe-Fmr1, si-NC, si-Fmr1, oe-Fmr1 + NC-mimic, and oe-Fmr1 + miR-335-3p mimic. **g** The quantitative analysis for number and size of neurospheres. **h** NSC cell cycle assayed by flow cytometry. **i** The expression of self-renewal-, differentiation-, and p53 signaling pathway-related genes in NSCs measured by RT-qPCR. **j** The protein level of self-renewal-, differentiation-, and p53 signaling pathway-related genes (p53, p21) in NSCs determined by Western blot analysis. The data were measurement data. Unpaired *t* test was employed to analyze the difference between two groups, while differences among multiple groups were analyzed using one-way ANOVA, with Tukey’s post hoc test. The statistical results were described as mean ± standard deviation. *p < 0.05 versus NSCs without any induced-differentiation treatment, NSCs treated with oe-NC, si-NC, or oe-Fmr1 + oe-NC. The experiment was performed three times

We furthermore explored the downstream target gene of miR-335-3p and the mechanism by which miR-335-3p promotes NSC self-renewal. It has been reported previously [23] that FMR1 is related to the differentiation of neural stem cells. The bioinformatics website (http://www.targetscan.org) predicted that Fmr1 was one of the target genes of miR-335-3p (Fig. 5a). Therefore, we assumed that miR-335-3p could target Fmr1 to affect NSC self-renewal. In an attempt to verify this prediction, RT-qPCR and Western blot analysis documented that Fmr1 indeed had high expression during NSCs differentiation (Fig. 5b). Besides, when miR-335-3p mimic was introduced into NSCs, Fmr1 expression was reduced, which was opposite to the results upon treatment of miR-335-3p inhibitor (Fig. 5c). Next, the targeting relationship between miR-335-3p and Fmr1 was verified through dual-luciferase reporter assays. As depicted in Fig. 5d, the luciferase activity of Fmr1 WT was distinctly inhibited by miR-335-3p mimic, while the luciferase activity of Fmr1 MUT was unchanged. Meanwhile, we conducted RNA pull-down assay where the biotinylated RNA probe was incubated with RNA-protein complex, which bound to the magnetic beads. Results clearly showed that the miR-335-3p probe specifically enriched the expression of Fmr1 mRNA (Fig. 5e), indicating that miR-335-3p could target to Fmr1 3′-UTR. RT-qPCR results demonstrated that, relative to the treatment with oe-Fmr1 + NC-mimic, Fmr1 expression was diminished after treatment of oe-Fmr1 + miR-335-3p-mimic (*p* < 0.05; Fig. 5f).

To investigate the effect of miR-335-3p/Fmr1 on NSC self-renewal and differentiation, we carried out neurosphere formation assay. According to the results, the number and size of neurosphere and NSC proliferation were increased in response to miR-335-3p overexpression, the effects of which could be abolished by Fmr1 overexpression. However, the number and size of neurospheres and NSC proliferation rate were enhanced when Fmr1 was silenced (Fig. 5g, h). Next, expression of self-renewal-related, differentiation-related, and p53 signaling pathway-related genes (p53 and p21) was measured using RT-qPCR and Western blot analysis. Results showed that miR-335-3p overexpression resulted in increased expression of Nestin, Msi1, Hes1, and Bmi1 expression and decreased expression of Nf-M, Tuj-1, GFAP, CNPase, p21, and phosphorylated p53, which were completely opposite to corresponding findings after silencing Fmr1. Besides, the alteration in the expression of these genes caused by Fmr1 overexpression alone was reversed in response to the combination of Fmr1 and miR-335-3p overexpression (Fig. 5i, j). It could thus be concluded that miR-335-3p downregulated Fmr1, thereby repressing NSC differentiation and promoting NSC self-renewal as well as inactivating p53 signaling pathway.

### FoxM1-activated miR-335-3p suppressed p53 signaling pathway by inhibiting Fmr1 to maintain NSC self-renewal and repress NSC differentiation

**Fig. 6:**
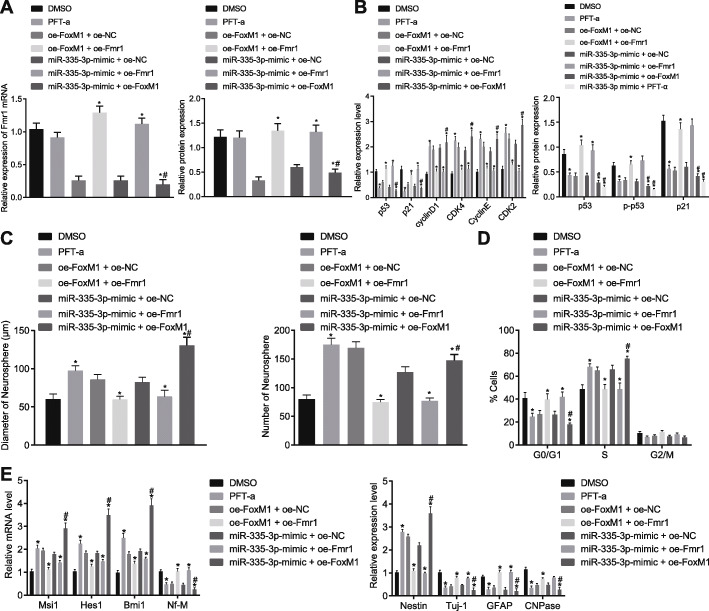
NSC self-renewal and differentiation were regulated by FoxM1-mediated miR-335-3p/Fmr1/p53 signaling pathway. NSCs were treated with DMSO, PFT-α, oe-FoxM1 + oe-Fmr1, miR-335-3p mimic + oe-Fmr1, oe-FoxM1 + oe-NC, miR-335-3p mimic + oe-NC, or miR-335-3p mimic + oe-FoxM1. **a** Fmr1 expression in NSCs detected by RT-qPCR and Western blot analysis. **b** Expression of p53 signaling pathway-related genes in NSCs determined using RT-qPCR and Western blot analysis. **c** The quantitative analysis for number and size of neurospheres. **d** NSC cell cycle measured by flow cytometry. **e** Expression of self-renewal-related and differentiation-related genes in NSCs examined by RT-qPCR. The measurement data were expressed as mean ± standard deviation. Differences among multiple groups were analyzed by one-way ANOVA, followed by Tukey’s post hoc test. *p < 0.05 versus treatment of DMSO, oe-FoxM1 + oe-NC or miR-335-3p mimic + oe-NC. The experiment was carried out in triplicate

Since the aforementioned experiments have elucidated the upstream and downstream regulatory network of miR-335-3p, our next step was to explore the combined effect of FoxM1/miR-335-3p/Fmr1 on NSC self-renewal and differentiation. Results from RT-qPCR and Western blot analysis demonstrated that the p53 signaling pathway was inhibited by treatment with PFT-α, an inhibitor of p53 [24], but that Fmr1 expression was hardly affected, indicating that p53 might act downstream of Fmr1 (*p* > 0.05). However, when NSCs were treated with oe-FoxM1 + oe-NC, miR-335-3p mimic + oe-NC, Fmr1 expression was distinctly reduced, which was then reversed by the transfection of oe-FoxM1+ oe-Fmr1 or miR-335-3p mimic + oe-Fmr1, but was further promoted by treatment of miR-335-3p mimic + oe-FoxM1 (Fig. 6a). Meanwhile, RT-qPCR and Western blot analysis were conducted for determining the expression of p53 signaling pathway-related genes. Compared with DMSO vehicle, PFT-α significantly suppressed the p53 signaling pathway, as demonstrated by diminished expression of p21 and phosphorylated p53. Surprisingly, overexpression of FoxM1 or transfection of miR-335-mimic also inactivated the p53 signaling pathway, while the combination of miR-335-3p mimic and oe-FoxM1 or PFT-α exerted a greater inhibitory effect on p53 expression in NSCs. The addition of oe-Fmr1 reversed the effect of oe-FoxM1 or miR-335-3p mimic, thus activating p53 signaling pathway and increasing expression of p21 and phosphorylated p53 (*p* < 0.05; Fig. 6B). These lines of evidence revealed that FoxM1 inhibits p53 signaling pathway through activation of miR-335-3p to decrease Fmr1 expression.

Moreover, NSC self-renewal and differentiation were observed in the absence or presence of oe-FoxM1, miR-335-3p mimic, or oe-Fmr1. Based on neurosphere formation assay and flow cytometry results, PFT-α treatment increased the number and size of neurosphere and cell proliferation, but opposite effects were seen upon treatment with oe-FoxM1 + oe-Fmr1 or miR-335-3p mimic + oe-Fmr1 in comparison to oe-FoxM1 + oe-NC or miR-335-3p mimic + oe-NC, respectively (*p* < 0.05). Furthermore, the number and size of neurospheres and cell proliferation rate were increased to a much greater extent after the transfection of miR-335-3p mimic + oe-FoxM1 than after treatment of miR-335-3p mimic + oe-NC (*p* < 0.05; Fig. 6c, d). Then, RT-qPCR results presented that Nestin expression was elevated while Tuj-1, GFAP, and CNPase expression was reduced after PFT-α treatment, and the opposite effects were found to be exerted by the overexpression of FoxM1 or Fmr1 alone or their combination, or the combination of miR-335-3p and Fmr1 overexpression. Besides, the alteration in the expression of these genes was more conspicuous in response to the combination of miR-335-3p and FoxM1 overexpression as compared with miR-335-3p overexpression alone (Fig. 6e). Taken together, these results illustrated that FoxM1-activated miR-335-3p decreased Fmr1 expression to suppress NSC differentiation and stimulate NSC self-renewal by inactivating the p53 signaling pathway.

## Discussion

There is well-documented evidence demonstrating the regulatory role of miRNAs in NSCs, highlighting the significance of the present investigation of the effect of miRNAs on NSC differentiation and self-renewal. For example, overexpressing miR-346 inhibited NSC proliferation [25]. Besides, another study also showed that downregulation of miR-302 family occurred during neural differentiation [26]. These findings suggest that the miRNA regulatory network may be a desirable target for research into possible neuroprotective strategies. Therefore, we conducted this study of the specific role of FoxM1-mediated effects on the miR-335-3p/Fmr1/p53 signaling pathway in NSC self-renewal. Our key results illuminated that FoxM1-activated miR-335-3p diminished Fmr1 expression to promote NSC self-renewal and to inhibit NSC differentiation by blocking the p53 signaling pathway.

Many transcription factors play important roles during adult neurogenesis, but essential differences exist in the biological responses of neural precursors [27]. Changes in transcription factor expression are also a way of modulating the response and consequent generation, dependent on non-coding RNAs (ncRNAs), mainly the miRNAs [28]. Some miRNAs are involved in determining the fate of NSCs, cell survival and maturation of dentate gyrus neurons, and other miRNAs in neurons/glia in the adult hippocampus [29]. Our present findings showed that transcription factor FoxM1 expression was diminished during NSC differentiation, while overexpressing FoxM1 could maintain NSC self-renewal and suppress NSC differentiation by activating miR-335-3p. A prior study presented findings of upregulation of FoxM1 in NSCs [22]. Besides, FoxM1 has been indicated to promote other miRNAs, including miR-130b, miR-301a, and the miR-15-16 and miR-17-92 clusters, and the interaction of FoxM1-miRNA participates in the regulation of NSC self-renewal [11]. During tumorigenesis, FoxM1 expression increases as cells differentiate from neural progenitor cells to pretumorigenic progenitors, and then to glioma stem-like cells [30]. Moreover, FoxM1 is involved in brain development as well as self-renewal of glioma stem cells [31]. It seems that FoxM1 suppresses NSC differentiation and promotes self-renewal by upregulation of miRNAs, and also participates in glioma, such that its effects on specific neurological and oncological conditions merit further investigation. The effect of FoxM1 on NSCs may be not direct, but could depend on its regulation of miR-335-3p expression and related signaling pathways.

Furthermore, our study pointed out the expression of miR-335-3p was downregulated during NSC differentiation. Previous evidence has uncovered that miR-335-3p participated in the process of neurological deficits and neuronal injury [32], and identified its diminished expression during the period of NSCs differentiation, which matches with our present results. Subsequently, we altered miR-335-3p expression to see how this influenced NSC differentiation and self-renewal. Based on prior work, we expected that miRNAs could influence NSC differentiation or self-renewal [33]. In the present study, we proved that ectopic expression of miR-335-3p repressed NSC differentiation but enhanced NSC self-renewal. Moreover, another study also identified that the canonical Wnt signaling pathway enhanced miR-335 expression in human mesenchymal stem cells, which was a positive regulator of MSC self-renewal [34].

Another interesting result of our study was that miR-335-3p stimulated NSC self-renewal and repressed NSC differentiation by inactivating the p53 signaling pathway via Fmr1. According to the previous literature, miR-130b targeting Fmr1 mediated the proliferation and differentiation of embryonic neural progenitor cells [35]. A study conducted by Gong et al. indicated that Fmr1 was a direct target gene of miR-335-5p [12]. In mouse NSCs, Fmr1 knockdown led to significantly diminished content of p53 [14]. Our study revealed that the p53 signaling pathway was positively regulated by Fmr1. Previously, an interaction between p53 and Fmr1 was implicated in the defective p53 signaling and dysregulated cell cycle control in Fragile X syndrome induced by silencing of Fmr1 [36]. Inhibiting p53 in Fmr1 knockout cultures restores the synchronization of neural network activity and partially corrects the homeostatic reductions of neural network integrity [37]. Meanwhile, Hou et al. also observed that the activation of p53-p21 signaling pathway could inhibit the self-renewal of mouse NSCs [15]. A recent study also revealed that the self-renewal of normal mouse NSCs was repressed by stimulating the activation of Lkb1-p53-p21 signaling pathway [15]. Besides, our data revealed that FoxM1 overexpression could enhance the inhibitory effect of miR-335-3p overexpression on p53 expression. In relation to this, accumulating evidence has illuminated that FoxM1 may inactivate the p53 signaling pathway by regulating microRNAs-mediated Fmr1 [11, 35] or other downstream targets of p53 signaling pathway such as Trp53inp1 [35, 38]. Taken together, NSC self-renewal and differentiation were affected by FoxM1-driven miR-335-3p/Fmr1/p53 signaling pathway.

## Conclusions

**Fig. 7:**
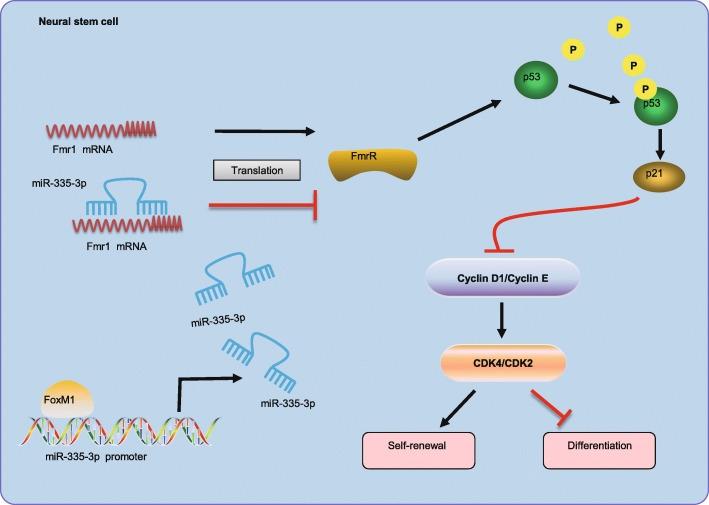
The mechanism graph of the regulatory network and function of FoxM1-mediated miR-335-3p in NSC self-renewal. miR-335-3p activated by FoxM1 could suppress NSC differentiation but promote NSC self-renewal by inactivating p53 signaling pathway via binding to Fmr1

Overall, the obtained data from our study provide new evidence about the mechanism of FoxM1-mediated miR-335-3p expression in maintaining the self-renewal and inhibiting the differentiation of NSCs by inactivation of the p53 signaling pathway via Fmr1 (Fig. 7). Our study may provide a potential strategy for controlling the fate of NSCs, which might eventually have therapeutic potential. However, there remains much to be learned about the regulatory mechanisms of the manipulation of miR-335-3p activation in NSCs.

## Data Availability

The datasets generated/analyzed during the current study are available.

## Abbreviations

NSCs: Neural stem cells
EGF: Epidermal growth factor
DMSO: Dimethyl sulfoxide
PBS: Phosphate buffer saline
CCK-8: Cell Counting Kit-8
ChIP: Chromatin immunoprecipitation
